# Supplementary material for: Assessing the effect of the COVID-19 pandemic, shift to online learning, and social media use on the mental health of college students in the Philippines: A mixed-method study protocol
Source: PLoS One. 2022 May 3;17(5):e0267555. doi: 10.1371/journal.pone.0267555 (PMC9064082; doi:10.1371/journal.pone.0267555)
Supplement: Attachment — Submitted filename: Response to Reviewers.docx [file pone.0267555.s001.docx]

**PONE-D-21-17998:** Assessing the Effect of the COVID-19 Pandemic, Shift to Online Learning, and Social Media Use on Mental Health Among College Students in the Philippines: A Mixed-Method Study Protocol

**Reviewer No. 1:**

This study protocol aims to assess the psychological effects among college students (18-25 years old) in the Philippines from the global pandemic, COVID-19, shift to online learning, and social media usage. The objectives of the study protocol address using a mixed-method study design that utilizes the quantitative and qualitative components. For the quantitative analysis, the authors propose sending an online self-administered questionnaire to the eligible participants to answer on socio-demographic factors. Based on the information provided, mental health outcomes will be assessed using two validated survey tools, Depression, Anxiety, and Stress Scale (DASS-21) and Brief-COPE. Additionally, the authors propose estimating the association between mental health outcomes and possible risk factors by using generalized linear models. Key informant interviews, a part of the qualitative component that addresses the stressors affecting the student’s mental health and behavior during the quarantine period. Finally, the authors suggest evaluating the data from quantitative and qualitative sources by using data triangulation, which analyzes multiple sources of data to enhance the credibility of a research study.

The careful methodology provided in this study protocol will allow other researchers to apply this design to their studies. The validation of this study should provide a roadmap to study the effect of the pandemic on students in other countries.

The design of the study is very detailed for the most part. The authors have provided the necessary information about how the study population would be recruited and provided a justification for the sample size (quantitative data) that would be included in the study by providing relevant power calculations. However, for the qualitative study, increasing the number of participants from different areas of the country would improve the quality of the outcome. Also, I would like to ask if any of the authors are Psychologists? If not, please acknowledge the Psychologists if the authors received any help in designing the study.

*Response: One of the authors is a senior consultant psychiatrist, Dr. Josefina T. Ly-Uson. She helped in the design of the study.*

Also, the inclusion and exclusion criteria of the study population could be explained in more detail. It is not clear **if only currently enrolled students would be included in the study**. It would be important to **justify the exclusion criteria**.

*Response: The inclusion criteria now included “currently enrolled college students.” The exclusion criteria included those students in graduate-level programs and those whose current curricula involve going on duty. We purposely chose to exclude those students because they have a different set of schooling conditions compared to the rest of the regular college students.*

Acknowledging similar studies (Copeland et al., 2021 and Fawaz et al., 2021) would help readers with a greater context. I would like to suggest the authors cite the peer-reviewed version of the article titled “Barriers to online learning in the time of COVID-19: A national survey of medical students in the Philippines”. Also, I would like to ask the authors to change the references according to the journal requirements and have a uniform style.

*Response: Thank you for the suggestion. This comment is noted, and revisions have been made.*

**Reviewer No. 2**

Thank you for the opportunity to review the study protocol. The protocol is for a mixed-methods study looking at the impact of COVID19 and the subsequent shift to quarantine (stay at home), the implementation of online learning formats, and social media use on college students’ mental health. The study will provide insight into factors impacting mental health of college students. There are some issues with the study protocol that should be addressed.

Introduction

Paragraph 2.

• If you refer to SARS-CoV-2 as COVID-19, please include swine flu when referring to H1N1.

*Response: This is noted, and revisions have been made.*

Paragraph 3.

• It is not clear which pandemic the authors are referring to.

*Response: This is noted, and revisions have been made.*

• Include reference examples for the first sentence.

*Response: This is noted, and revisions have been made.*

• Provide examples of how infection and death have "adversely affected" mental health.

*Response: This is noted, and revisions have been made.*

• How many people responded to the survey measuring the psychological effects of COVID19 in the Philippines?

*Response: This is noted, and revisions have been made.*

Paragraph 4.

• Provide references for the first three sentences.

*Response: This is noted, and revisions have been made.*

• Provide details of the studies you reference.

*Response: This is noted, and revisions have been made.*

• Final sentence is conflating being infected by COVID19 and suffering from mood and substance disorders - please clarify exactly what is meant.

*Response: This is noted, and revisions have been made.*

Paragraph 5.

• University students are not generally accepted as a vulnerable population. Please provide a reference that supports this statement.

*Response: This is noted, and the statement was deleted.*

• Second sentence - provide the references for the multiple studies.

*Response: This is noted, and revisions have been made.*

• Are Chinese university students similar to Filipino college students? Surely there are other studies from other countries that can be included here. Or is the social, cultural, and political situation similar between the Philippines and China?

*Response: This is noted, and revisions have been made. We cited prevalence rates from three different countries (USA, Malaysia, and China).*

Paragraph 6.

• Second sentence is not clear. Do you mean that academic performance is associated with student mental health? If so, just say that.

*Response: This is noted, and revisions have been made.*

• What is "this" in the sentence: "Online learning poses multiple challenges to this".

*Response: This is noted, and revisions have been made.*

• Provide an example of "Students’ various social support systems" that have to adapt.

*Response: This is noted and revisions have been made.*

• In this sentence: "These challenges are alarming because social support has been noted as a critical aspect of mediating acute distress disorder" it's not clear if the statement refers to students or some other population.

*Response: This is noted, and revisions have been made.*

• References are needed for the following sentences: "In addition, loneliness has been rising for the past six years amongst this vulnerable demographic. One study showed that being a student is a risk factor for loneliness, exacerbated during the pandemic." Furthermore, please provide greater clarity around the population being discussed.

*Response: This is noted, and revisions have been made.*

• The following sentence does not follow the logic from the preceding sentences: "Therefore, online learning must be perceived as an inclusive community and a safe space for peer-to-peer interactions (18)."

*Response: This is noted, and revisions have been made.*

Paragraph 7.

• The following sentence needs a reference: "One research recommends clear and focused design elements on accommodating students living with depression."

*Response: This is noted, and revisions have been made.*

• The argument presented in this paragraph is not clear.

*Response: This is noted, and revisions have been made.*

Paragraph 8

• The first three sentences need references.

*Response: This is noted, and references have been added.*

• The argument for examining the effect of social media on students' mental health is weak and is only presented in the second to last paragraph.

*Response: This is noted, and revisions have been made.*

Paragraph 9

• There is no inclusion of social media in this paragraph.

*Response: This is noted, and revisions have been made.*

• This paragraph should present a strong argument for the study, including all the factors that are to be included in the study.

*Response: This is noted, and revisions have been made.*

Materials and methods

Study aim

• Aims 1 and 2 are very similar. Aim 1 suggests you are going to describe the sample according to the categories of mental health. This is quite unusual and makes me think that maybe 'stratified' is not the correct word for this aim. Perhaps what is meant is that the study aims to describe the characteristics of the sample population including mental health (e.g., depression, anxiety, stress)

*Response: This is noted. We replaced the word stratified with categorized. Aims 1 and 2 are not similar. The first aim will present the characteristics of the population, while the second aim will present the prevalence and risk factors.*

• Aim 2 it is not clear if the determination of prevalence is before or post, the subsequent shift to online learning. Please clarify.

*Response: Aim 2 will determine the prevalence after the subsequent shift to online learning. We will not determine the pre-shift to online learning prevalence because there was an abrupt transition to online learning in the Philippines, which limits our time frame to collect data before the shift to online learning.*

• Aim 3. It is not clear what the aim is. Please simplify. It might require breaking this one aim up into 2 or 3.

*Response: Aim 3 was split into 2 aims. The first aim will be looking at the effect of social media use on markers of mental health (depression, anxiety, stress, and coping strategies towards stress). At the same time, the second aim will look at the effect of online learning shift on markers of mental health (depression, anxiety, stress, and coping strategies towards stress).*

• Aim 4 Is similar to the last aspect of aim 3.

*Response: Aim 4 is very different from Aim 3 because Aim 4 is the qualitative part of the study which will use key informant interviews to explore facilitators or stressors that modified the mental health status of the participants while Aim 3 is the quantitative part.*

• Furthermore, the phrase "during the COVID-19 pandemic, quarantine, and subsequent shift to online learning." Is confusing - COVID 19 is current, it's not clear whether all or some of the students are in quarantine, and presumably, they have shifted to online learning (past). Please clarify the state in which the study will be conducted.

*Response: Currently, the Philippines is still in quarantine due to COVID-19 and all classes made a shift to online learning and no face-to-face classes are allowed.*

Design

• When is the quarantine period? Please provide dates?

*Response: Currently, the Philippines is still in quarantine. We opted to use April to November 2021 as the study period.*

• Population RCY seems like a great way to recruit participants. However, this population may not reflect all college students as RCY are volunteers. Students who volunteer may have different values and attitudes towards mental health, and social justice and adapting to change. The authors must account for this in their study and ensure there are no differences between their RCY participants and the non-member RCY participants on critical factors (e.g., mental health etc).

*Response: RCY is connected with the majority of the universities and colleges in the Philippines. RCY will spearhead the distribution of the questionnaires to universities and colleges in the Philippines. Therefore, non-RCY volunteers are also encouraged and recruited to participate in the study. We will account for the differences between RCY and non-RCY volunteers by adding an indicator variable in our statistical models.*

• It is not clear how random sampling for the KII will be achieved. The reported sampling method reads more like stratified sampling.

*Response: Yes, it is a stratified random sampling using 4 major regions of the country. We have revised the section.*

Inclusion exclusion criteria

• It is not clear why those who identify as non-binary genders are not included. Why is gender an inclusion/ exclusion criterion?

*Response: Gender is not an exclusion/inclusion criterion. Revisions have been made.*

• The sample size calculation - how was the number of KIIs determined? how data saturation will be determined

*Response: The saturation will be achieved once almost all of the interview transcripts have been generating no new information. We added the reference Hagaman and Wutich as basis for the KII sample size.*

Study procedure

• It's not clear how the demographic factors will be collected.

*Response: Demographic factors will be collected using the SAQ we developed.*

• Variables are not clear (e.g., sickness of loved ones) - do you mean family members? friends? pets? do you mean chronic illness or acute illness?

*Response: This is noted and revisions have been made.*

• What other factors that could affect mental health are you going to measure?

*Response: We deleted “other factors that could affect mental health”.*

• This statement is not accurate: "The DASS-21 will measure the prevalence of depression, anxiety, and stress-related issues affecting daily life (28)" please correct to more accurately describe the DASS.

*Response: This is noted and revisions have been made.*

• It is not clear how this variable will be measured: "the total time spent on social media (TSSM) per day will be ascertained by querying the participants to provide an estimated time spent daily on social media during and after their online classes." is it the total time in one day, or only during and after class. Why not all day? or why is it only during and after interest? The assumption is that they will increase their use of social media, but what if their use of social media is the same or less than before the shift to online learning?

*Response: We will measure the social media during and outside online class hours. TSSM will measure the total time spent per day on social media. We will not account for the TSSM before shifting to online learning. Our objective is to measure their TSSM during and outside of class hours that is happening in a quarantine period.*

• Regarding the KII, it may be country-specific, but it's not clear how social scientists and research assistants facilitation of interviews will be the same quality as psychologists and psychiatrists. How will the difference in skills in interviewing be overcome?

*Response: Dr. Uson, our board-certified psychiatrist, trained the research assistants to facilitate KIIs. She will also be present during the KII. In the Philippines, training of the research assistants to facilitate KII is done in order for them to learn and be adequately trained for future research. Research assistants may someday be the research primary investigators.*

• Will the results of the survey be used to develop interview questions?

*Response: The results of the survey will not be used to develop interview questions. But we will use the answers of the KII participants in the survey as a guide during the interviews of the KII participants.*

Data Analysis

• Given that all the variables are known, the quantitative analysis could be clearer with examples of what the authors mean by 'covariate' and 'possible risk factors.

*Response: Yes, all the possible variables that will be used in the data analysis have been identified a priori as confounding factor covariates and risk factors.*

• Will the analyses be explorational? The literature review implies that some hypotheses may be developed. If so, the analyses should be designed to test those hypotheses.

*Response: The analysis will not be explorational. Important possible risk factors (exposures) and covariates (confounding factors) were chosen a priori and will be used for the multivariable generalized linear models.*

• How will p-values be adjusted to account for the multiple analyses?

*Response: We will not use a correction method to adjust the p-values, since our statistical analyses will be not that many to warrant an adjustment. Possible adjustments of p-values are for microarray datasets to correct the occurrence of false positives in the multiple analyses.*

Qualitative data

• Will the themes be developed independently by researchers? How many researchers will be involved in the coding? It is not clear from the description how triangulation will be established. Will multiple authors do the coding of the interview transcripts - independently? Will the results of the survey be used to inform the coding of the interviews?

*Response: This is noted, and revisions have been made. The description of the triangulation was described in the “data triangulation” section and references have been updated to include the “comparison of datasets for convergence”. As mentioned earlier, the results of the survey will not be used to develop interview questions. But we will use their answers in the survey as a guide during the interview.*

Ethics and dissemination

• It is still not clear how random sampling will be achieved by the authors for recruitment for KII.

*Response: From the pool of participants per area, using a software, we will randomly choose prospective KII participants and contact them if they are willing to participate. If they opted not to participate, we will randomly choose participants again from the pool of respondents per area.*

• What will the researchers do if a participant's response to the DASS indicates they have clinical levels of Depression, Anxiety or Stress?

*Response: It is our ethical duty to assist the participants if they have been screened to have high clinical levels of depression, anxiety, or stress. We will refer them to a tertiary government hospital for further evaluation, or treatment if needed.*

Discussion

• References are needed throughout the discussion. For example, "The role of social media is also an important exposure to some college students. Social media exposure to COVID-19 may be considered a contributing factor to college students’ mental well-being, particularly their stress, depression, and anxiety."

*Response: This is noted, and references have been added.*
