# Supplementary material for: Assessing the effect of the COVID-19 pandemic, shift to online learning, and social media use on the mental health of college students in the Philippines: A mixed-method study protocol
Source: PLoS One. 2022 May 3;17(5):e0267555. doi: 10.1371/journal.pone.0267555 (PMC9064082; doi:10.1371/journal.pone.0267555)
Supplement: Attachment — Submitted filename: Response to Reviewers_Round 2_Plos One 22022022.docx [file pone.0267555.s002.docx]

**RESPONSE TO REVIEWERS**

**PONE-D-21-17998:** Assessing the Effect of the COVID-19 Pandemic, Shift to Online Learning, and Social Media Use on Mental Health Among College Students in the Philippines: A Mixed-Method Study Protocol

Reviewer #1: Appreciate the authors' efforts, they addressed all the concerns adequately. However, I suggest authors cite the peer-reviewed version of the article instead of medRxiv.

Good luck with your publication.

**Response: We have removed the medRxiv references.**

Reviewer #2: Thank you for the opportunity to review this protocol. The authors have made considerable changes to the protocol in response to reviewer comments. There are just a couple of minor points to be addressed.

Paragraph 6 – please include the years that these studies were conducted. Also, as these studies are cross-sectional, it is not entirely accurate to say that there is a growing prevalence (which you could do if there were similar studies conducted years apart on the same population, or a longitudinal study. But these are separate populations and the years have not been presented to the reader). Perhaps just delete ‘growing’

**Response: We have deleted the word “growing.”**

Qualitative data – paragraph 1- The description of the qualitative data is missing some information. For example, at what point will the three qualitative analysts discuss the themes? Will the three analysts do all of the transcripts or will they do a sample to establish consistency before dividing the transcripts? Please confirm whether inductive or deductive logic to the coding approach.

**Response: The qualitative data description was added with information regarding the discussion of thematic analysis of the three researchers. To ensure consistency, KII training, including transcription and quality assurance, will be done with the research team members as detailed in the Data Management Plan Section. Moreover, an inductive logic approach to coding will be conducted. All of these are reflected in the revised manuscript.**

Reviewer #4: -The study is proposed to explore the effect of social media use, online learning on mental health. It is hard to differentiate whether the effect is due to social media or online learning. (Aim 3 and Aim 4).

**Response: Generalized linear model using Poisson regression will be done to independently analyze the effect of social media usage or online learning on the participants' mental health. Thus, social media usage and online learning will be treated as two exposures independently.**

- The study participants (inclusion criteria) also include out-of-school youth as they are including all RCY who agreed.

**Response: We have revised the inclusion and exclusion criteria. “Out-of-school youth” classification is now included in the exclusion criteria. Moreover, current enrollment in a university is part of the inclusion criteria.**
